# Novel Tissue Level Effects of the *Staphylococcus aureus* Enterotoxin Gene Cluster Are Essential for Infective Endocarditis

**DOI:** 10.1371/journal.pone.0154762

**Published:** 2016-04-28

**Authors:** Christopher S. Stach, Bao G. Vu, Joseph A. Merriman, Alfa Herrera, Michael P. Cahill, Patrick M. Schlievert, Wilmara Salgado-Pabón

**Affiliations:** Department of Microbiology, University of Iowa Carver College of Medicine, Iowa City, IA, 52242, United States of America; Instituto Butantan, BRAZIL

## Abstract

**Background:**

Superantigens are indispensable virulence factors for *Staphylococcus aureus* in disease causation. Superantigens stimulate massive immune cell activation, leading to toxic shock syndrome (TSS) and contributing to other illnesses. However, superantigens differ in their capacities to induce body-wide effects. For many, their production, at least as tested *in vitro*, is not high enough to reach the circulation, or the proteins are not efficient in crossing epithelial and endothelial barriers, thus remaining within tissues or localized on mucosal surfaces where they exert only local effects. In this study, we address the role of TSS toxin-1 (TSST-1) and most importantly the enterotoxin gene cluster (*egc*) in infective endocarditis and sepsis, gaining insights into the body-wide versus local effects of superantigens.

**Methods:**

We examined *S*. *aureus* TSST-1 gene (*tstH*) and *egc* deletion strains in the rabbit model of infective endocarditis and sepsis. Importantly, we also assessed the ability of commercial human intravenous immunoglobulin (IVIG) plus vancomycin to alter the course of infective endocarditis and sepsis.

**Results:**

TSST-1 contributed to infective endocarditis vegetations and lethal sepsis, while superantigens of the *egc*, a cluster with uncharacterized functions in *S*. *aureus* infections, promoted vegetation formation in infective endocarditis. IVIG plus vancomycin prevented lethality and stroke development in infective endocarditis and sepsis.

**Conclusions:**

Our studies support the local tissue effects of *egc* superantigens for establishment and progression of infective endocarditis providing evidence for their role in life-threatening illnesses. In contrast, TSST-1 contributes to both infective endocarditis and lethal sepsis. IVIG may be a useful adjunct therapy for infective endocarditis and sepsis.

## Introduction

*Staphylococcus aureus* causes life-threatening infections. In the U.S. yearly, *S*. *aureus* accounts for approximately 5,000 cases of toxic shock syndrome (TSS) [1, 2], 70,000 cases of pneumonia [1, 3], 40,000 cases of infective endocarditis (IE) [3–6], and more than 500,000 post surgical infections [3, 7]. *S*. *aureus* is the leading cause of IE and the second leading cause of sepsis [3, 8]. Staphylococcal enterotoxins (SEs), SE-*like* (SE*l*) molecules, and TSS toxin-1 (TSST-1 encoded by *tstH*) are secreted virulence factor superantigens (SAgs). SAgs are present in all pathogenic strains and are critical in *S*. *aureus* infections [1, 9–13].

SAgs crosslink T lymphocytes and antigen-presenting cells causing massive cytokine production, contributing to diseases through immune system dysfunction, with SAg lethal effects dependent on direct toxic and cytokine effects on the cardiovascular system [14, 15]. Cytokines that are induced include IL-1β, IL-6, TNF-α and TNF-β [1]. Humans are sensitive to SAgs, showing hypotension and fever at doses as low as 0.001 μg/kg [16]. Likewise, rabbits are sensitive to the toxic effects of SAgs, whereas mice are highly resistant [17]. Yet, of 25 SAgs, only TSST-1, SEB, and SEC are associated with TSS [1, 18]. When encoded, these SAgs are produced at levels high enough to become systemic as evidenced by milligrams per milliliter being produced *in vitro* in biofilms [19] and up to 100 μg/ml in tampons in women [1, 10, 20, 21]. This suggests other SAgs, produced at nanogram and picogram per milliliter concentrations [10] remain at infection sites, where they exert local effects, contributing to tissue inflammation during disease progression.

**Fig 1:**
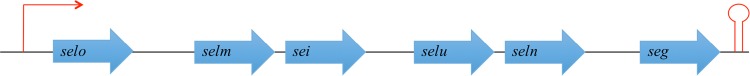
Arrangement of the *enterotoxin gene cluster (egc)* in *S*. *aureus*. The relative positions of the 6 SE and SE*l* genes, in blue, are located but not drawn precisely to scale. The putative promoter and transcriptional terminator are shown.

SAgs of the enterotoxin gene cluster (*egc*) are the most prevalent SAgs among *S*. *aureus* strains today [22–24]. The *egc* operon encodes up to 6 SAgs: SEG, SEI (sometimes referred also as SE*l*I, SE*l*M, SE*l*N, SE*l*O, and SE*l*U (a small minority encode two pseudogenes, φent1 and φent2, instead of SE*l*U) (Fig 1) [23]. The role of the *egc* SAgs in pathogenesis is unclear [25]. We used the rabbit model of life-threatening *S*. *aureus* infections (sepsis with IE) to determine the contribution of *egc* SAgs to *S*. *aureus* pathogenesis.

IE is the most severe of *S*. *aureus* diseases, occurring in 30–60% of patients with *S*. *aureus* bacteremia [4, 26]. The organism gains access to the circulation from skin/soft tissue infections, catheters, surgical wounds, and pneumonia [27, 28]. IE is an infection of the heart endothelium, predominantly valves with the characteristic cauliflower-like vegetations, that leads to debilitating complications and in-hospital mortality of 20–40% [29–31]. Life-saving interventions, such as valve replacement, cardiac devices, and hemodialysis, unfortunately increase the risk of IE [5]. Recent studies highlight the high prevalence of SAg genes encoding TSST-1, SEC, and *egc* SAgs in IE patients [32, 33]. Gene deletion and complementation studies in *S*. *aureus* strain MW2 provided evidence for the critical role of SEC in IE causation and disease severity, as tested in the rabbit model of native valve IE [10], demonstrating that SAg genes in IE strains represent causation rather than linkage disequilibrium with unknown factors, as initially suggested [32].

The studies presented herein addressed the contribution of *egc* SAgs and TSST-1 to both development of IE and lethal sepsis in the clinically relevant *S*. *aureus* strain MN8. We confirmed the unique association of lethal sepsis with TSST-1 and demonstrated its contribution to rapid progression of IE. Furthermore, we provide evidence of the critical contribution of *egc* SAgs in the pathophysiology of IE. Finally, we show that intravenous immunoglobulin (IVIG) combined with vancomycin prevents IE, stroke development, and lethal sepsis.

## Materials and Methods

### Ethics Statement

Rabbit research was performed with approval given by the University of Iowa Institutional Animal Care and Use Committee. The approved animal protocol number was 1106140 and then replaced after three years by new protocol number 4071100. Numbers of rabbits required for experimentation was determined by power analysis and past experience. Subcutaneous surgical procedures were used to cause damage to the aortic valves of rabbits according to the method we have used for more than 15 years [34]. The animals, approximately 50% male and 50% female, were anesthetized with the combination of ketamine 10 mg/kg) and xylazine (10 mg/kg) subcutaneously; they were monitored every 15 min for appropriate depth of anesthesia and appropriate breathing. Upon recovery from surgery, all rabbits were administered 0.05 mg/kg twice daily of buprenorphine for pain relief. SAgs are known to cause lethality, including through TSS and sepsis, both severe effects studied in this manuscript. We have experimentally determined over more than 40 years, that predictors of lethality due to *S*. *aureus* SAgs require development of shock in rabbits (This has been defined experimentally and by IACUC agreement as: 1) Failure of animals to exhibit typical wild animal escape behavior and 2) simultaneous failure to maintain upright positioning). The potential rapid development of shock in rabbits does not correlate well with weight loss and other measures of illness, but rather correlates with loss of fluid from the bloodstream. Thus, we use development of shock by the above agreed upon definition as the measure of 100% progression to death. When animals reached this point, they were prematurely euthanized with Euthasol (1 ml/kg). Thus, death was not used as an endpoint. However, due to some unpredictability in disease progression, sometimes very rapidly, 10% of animals succumbed to lethal shock without premature euthanasia. At the termination of experimentation, surviving animals were euthanized with Euthasol (1 ml/kg).

### Bacterial strains and growth conditions

*S*. *aureus* strain MN8 is a CC30 (USA200) strain isolated from a patient with menstrual TSS. The strain produces TSST-1 and has been reported to produce SEC by antibody reactivity, but this appears to be cross-reactivity with SE*l*U; the strain lacks the gene for SEC. By mass spectrometry [35], strain MN8 has been shown to produce detectable amounts of SE*l*U, but barely sufficient amounts for accurate quantification (the lower limit of our detection is approximately 75 pg/ml). Lyophilized stocks of low passage (two times subcultured from the original patient isolate) are maintained in the laboratory. *S*. *aureus* RN450 is a relatively avirulent laboratory strain derived from RN25 [36]. The strain does not produce SAgs, but produces cytotoxins and cell-surface virulence factors. *S*. *aureus* were grown in Todd-Hewitt (TH) broth (Becton Dickinson, Sparks, MD) at 37°C with aeration, to stationary phase, diluted, and washed in phosphate-buffered saline (PBS) before infection. *E*. *coli* were grown in Lysogeny Broth (LB) Miller formulation (10g/L Tryptone, 10g/L NaCl, 5g/L Yeast Extract) at 37°C with aeration. Strains harboring plasmids were maintained using erythromycin (10 μg/ml) or carbenicillin (100 μg/ml).

### Construction of *S*. *aureus* MN8 Δ*tstH*, MN8 Δ*egc*, and MN8 Δ*tstH*Δ*egc*

DNA was isolated using the Qiagen DNeasy Blood & Tissue Kit according to the manufacturer’s protocol for Gram-positive organisms. Plasmid DNA was isolated using the Qiagen QIAprep Spin Miniprep kit in accordance with manufacturer’s protocol. In-frame deletions of *tstH* and *egc* in the *S*. *aureus* MN8 strain were generated as described previously [10] using PCR products amplified with primers listed in Table 1. Deletions were introduced using allelic exchange with pJB38 [37] and verified by PCR and Western immunoblot. The *egc* was expressed in *S*. *aureus* RN450 from the pCE104 plasmid under the control of the *sec* promoter.

**Table 1:** Primers used to generate gene deletion constructs and in qRT-PCR to characterize expression of the *egc* in *Staphylococcus aureus* MN8.

Primer	Sequence 5'-3'
MN8tstHRT FWD Set 2	AGACTGGTATAGTAGTGGGTCTG
MN8tstHRT REV Set 2	TGATGCTGCCATCTGTGTT
gyrBRT FWD Set 1	TATGGTGCTGGGCAAATACA
gyrBRT REV Set 1	CCTCTCTCTGAAGTCGATCCTA
SElORT FWD Set 2	AGTGGAATTTAGCTCATCGGAA
SElORT REV Set 2	CTTGATGCTCACCATGACAATG
SElMRT FWD Set 1	CTGTTGATGTATACGGCCTAAGT
SElMRT REV Set 1	CACCCGCTAAAGTAACTCCTC
SEiRT FWD Set 1	GCAAGGAGATATTGGTGTAGGT
SEiRT REV Set 1	GATCAAATCATTGGTACCGGTTG
SElURT FWD Set 4	CAGCGGATAATATGGAATTAAATGATGG
SElURT REV Set 4	ATTTCCATCATGCTCGGTCAC
SElNRT FWD Set 1	CATGCTTATACGGAGGAGTTACG
SElNRT REV Set 1	ACCTTCTTGTTGGATACCATCTT
SEGRT FWD Set 1	GAATTCCCAACCCGATCCTAAA
SEGRT REV Set 1	CCTTCAACAGGTGGAGACATATAA
EcoRI-UpFT	ATCCTAGAATTCGCTCTCAGAC CTAA
UpRTOH	GCTAGCACGCGTGAAAGTGTTTGTTA
DnFT-OH	ACGCGTGCTAGCCTAATCCTCACTAA
AvaI-DnRT	ATCCTACCCGGGGATGAGATTGTATT
MN8egcFupLKO	AAAAAGAATTCAGATCGCGGGTTCGA TTCCCGTCG
MN8egcRupLKO	GCTAGCACGCGTTGCTTTACTCTTTTTTATAATATCCCTCCTTCAC
MN8egcFdnLKO	ACGCGTGCTAGCTAAGTATATTTGATATCACTATACAATCATAAAG
MN8egcRdnLKO	aaaaaGGTACCTGATTATATTGTTTCAGAAACAAAATCAC

### Preparation of His-tagged *egc* proteins and antisera

Sequences for each *egc* gene were identified in strain MN8 using NCBI blast. Predicted signal peptides were identified for each protein by use of SignalP 4.0 Server. Primers were designed to amplify each gene without signal peptide sequence. Genes were amplified using the Expand High Fidelity PCR System (Roche Diagnostics). The PCR products were cloned and expressed using the pTrcHis TOPO TA expression kit (Life Technologies), which adds an N-terminal hexahistidine tag to the proteins. *E*. *coli* TOP10 cells were transformed with the constructs. Bacteria were cultured in LB-Miller broth supplemented with carbenicillin (100 μg/ml), and each *egc* gene verified by sequencing.

Expression of the *E*. *coli* TOP10 strains containing the constructs for each protein were grown in terrific broth supplemented with carbenicillin (100 μg/ml) at 37°C with aeration to an absorbance at 600nm wavelength of 0.6, at which point the cultures were shifted to 20°C, protein expression induced with 0.5 mM isopropyl-beta-D-thiogalactoside (IPTG), and cells grown for 16 hrs. Cells were pelleted at 15,000 x g for 15 min at 4°C. Cells were resuspended in 25 ml Buffer A (20 mM Tris pH 7.9, 0.5 M NaCl, 20 mM imidazole, 5% glycerol) with complete mini-EDTA free protease inhibitor (Roche Diagnostics, IN) and lysed by 2 passages at 30,000 pounds per square inch pressure using a microfluidizer (Microfluidics, Westwood, MA). Lysates were clarified by centrifugation at 15,000 x g for 15 min at 4°C and passed through a 0.45 μm filter. The clarified lysate for each toxin was loaded into a superloop and passed through a 1 ml HisTrap HP column (GE Healthcare Life Sciences). The proteins were purified on an ÄKTApurifier FPLC system (GE Healthcare Life Sciences) using Buffer A and Buffer B (20 mM Tris pH 7.9, 0.5 M NaCl, 500 mM imidazole, 5% glycerol). Protein elutions corresponding to the ultraviolet peak observed during FPLC purification were separated on 4–20% gradient Tris-Glycine SDS-PAGE gels, stained with colloidal Coomassie, and the samples corresponding to the proteins of interest were pooled and dialyzed against 1 L of PBS 4°C, which was changed twice before final dialysis at 4°C overnight. Sample purity was assessed using colloidal Coomassie stained 4–20% gradient Tris-Glycine SDS-PAGE gels. The his-tagged *egc* proteins electrophoresed at ~35 KDa. Protein concentrations were determined using Bradford reagent (Bio-Rad). Proteins were stored at 4°C for short term (weeks), -20°C intermediate (months to year), and -80°C for long-term.

Antisera were generated in New Zealand white rabbits with use of 50 μg of each purified his-tagged *egc* protein in 50:50 PBS: Freund’s incomplete adjuvant. Immunizations, every two weeks for four injections were made in the napes of the necks. One week later, blood was collected from the rabbits, antisera collected, and sera tested by Western immunoblotting. *egc* proteins were quantified by a previously developed Western immunoblot procedure [38].

### Expression of *egc* proteins in *S*. *aureus* RN450

Primers were designed to amplify 350 bp upstream through the signal peptide sequence (SECPROM) and 169 bp downstream (SECTERM, including the stop codon of *S*. *aureus* MW2 *sec4*. The SECTERM primer contained a 5’ 25 bp sequence complementary to the 3’ region of the SECPROM product, and reverse SECTERM primer contained a 25 bp complementary sequence to the pUC18 region of plasmid pCE104 [39]. The SECPROM and SECTERM products were assembled by overlapping PCR, and the resultant construct was cloned into the pCE104 plasmid using EMPPCR [40]. The resulting plasmid was verified by sequencing, designated pCPTCSS, and used to overexpress the *egc* proteins.

Subsequently, primers designed to omit the signal peptide sequence of each *egc* protein were used to amplify the *egc* proteins from *S*. *aureus* MN8. The *egc* PCR products were inserted into pCPTCSS using EMPPCR [40], transformed into *E*. *coli* DH5α, and verified by sequencing. Overexpression constructs were cloned into *S*. *aureus* RN4220 and then transduced into RN450 as previously described [10], verified by *egc* gene specific PCR, and protein expression characterized by Western immunoblot.

### Identifying promoters within the intergenic regions of the *egc*

Intergenic regions of the *egc* corresponding to sequences between *selo* and *selm*, between *sei* and *selu*, and between *seln* and *seg* were amplified by standard PCR using primers designed to introduce *Hind*III and *Kpn*I restriction sites. The PCR products were gel purified using the QIAquick gel extraction kit (Qiagen, CA), digested with *Hind*III and *Kpn*I, and ligated into the pCM11 vector, which contains super folder green fluorescent protein (GFP). *E*. *coli* DH5α were transformed with the GFP constructs, verified by sequencing, and electroporated into RN4220. Strains were grown to stationary phase and GFP expression measured fluorometrically on a Tecan microplate reader (excitation at 490 nm and emission at 520 nm wavelength).

### Quantitative real-time polymerase chain reaction (qRT-PCR)

MN8 cultures were diluted to 4–7 x 10^4^ and 2 x 10^6^ CFUs/ml and samples collected for analysis at 3, 6, and 11 hr (for the 10^4^ dilution) and 2, 5, and 10 hr (for the 10^6^ dilution). Samples were treated with RNAlater and RNA isolated using the RNeasy Mini Kit (Qiagen) according to manufacturer’s protocol. cDNA was generated using the High-Capacity cDNA Reverse Transcription Kit (Applied Biosystems). qRT-PCR was performed using the Power SYBR Green PCR Master Mix (Applied Biosystems) with gene specific primers (Table 1) and the following conditions: 95°C for 10 min, 40 cycles at 95°C for 30 seconds, and 54°C for 1 min, followed by a dissociation curve. Two biological replicates were analyzed in triplicate and relative expression normalized to *gyrB*.

### Rabbit models

Rabbits included both sexes (approximately 60% male) and weighed 2–3 kg. For all experiments, bacterial strains were cultured overnight to stationary phase and washed in PBS before intravenous inoculation. In rabbits administered strains with plasmids, animals were treated with erythromycin to maintain plasmids, and maintenance of plasmids *in vivo* was determined by plating of vegetation homogenates onto erythromycin TH-agar plates, which showed colony growth indicating retention of plasmids.

The combined IE/sepsis model was performed [41], wherein rabbits were injected through the marginal ear veins with 3–4 x 10^8^ MN8 or 3–4 x 10^9^ RN450 CFUs in PBS after damage to the aortic valve with a hard plastic catheter that was then removed. The doses of *S*. *aureus* used were previously determined to assure vegetation development by 24 hr with increases in vegetation size occurring over the 4 day test period. Animals were monitored 4 times daily during experimentation for development of clinical strokes defined as development of limb paralysis requiring premature euthanasia.

In studies using combinations of vancomycin and human IVIG to reverse the course of IE/sepsis, the following protocol was used. Rabbits were allowed to develop IE/sepsis for 24 hr after intravenous injection of 2–4 x 10^8^ MN8 in PBS. This time period is sufficient to allow *S*. *aureus* to initiate vegetation formation on aortic valves (total vegetation size is typically 10–20 mg). At this time, animals were injected intravenously with PBS (control), vancomycin alone (20 mg; twice daily), or IVIG (5 ml once daily) plus vancomycin (20 mg; twice daily). The dose of IVIG reflects the human dose scaled down to the size of rabbits, but unlike in humans, the IVIG in rabbits was given daily to account for possible blood persistence differences due to the human origin of the IVIG. Animals were monitored at least twice daily for fever responses through use of rectal thermometers.

### Statistics

Lethality of MN8 strains was compared using Log-rank, Mantel-Cox Test. Vegetation and bacterial CFU counts between MN8 and Δ*egc*, Δ*tstH*, and Δ*tstH*Δ*egc*, were compared using one-way ANOVA and non-parametric, Kruskal-Wallis test. Vegetations from RN450 expressing *egc* or *tstH* were compared using one-way ANOVA and non-parametric, Kruskal-Wallis test. GFP expression and comparison of the capacity of the *egc* SAgs to induce vegetations was compared using the Student’s t-test and non-parametric Mann-Whitney test. Survival in the IVIG experiments was compared using the Log-rank Mantel-Cox test. Log-rank Mantel-Cox test was used to compare the various. Vegetation size, development of fever, and shock in the IVIG experiments was compared using one-way ANOVA (with Dunnett's multiple comparisons test) and unpaired Student’s t-test. The GraphPad Software (Prism) was used for statistical analyses.

## Results

### *S*. *aureus* MN8 infective endocarditis is dependent on both TSST-1 and *egc* SAgs

We hypothesized that both TSST-1 and the *egc* SAgs contribute to development of IE. Epidemiological studies previously demonstrated that 58–90% of IE isolates encode *egc* SAgs, 9–20% TSST-1, and 18–25% SEC [32, 33]. Human pathogenic *S*. *aureus* strains always encode for one or a combination of SAgs. We previously demonstrated the critical requirement of SEC for IE in *S*. *aureus* MW2, an isolate exceptional at causing IE in rabbits [10].

**Fig 2:**
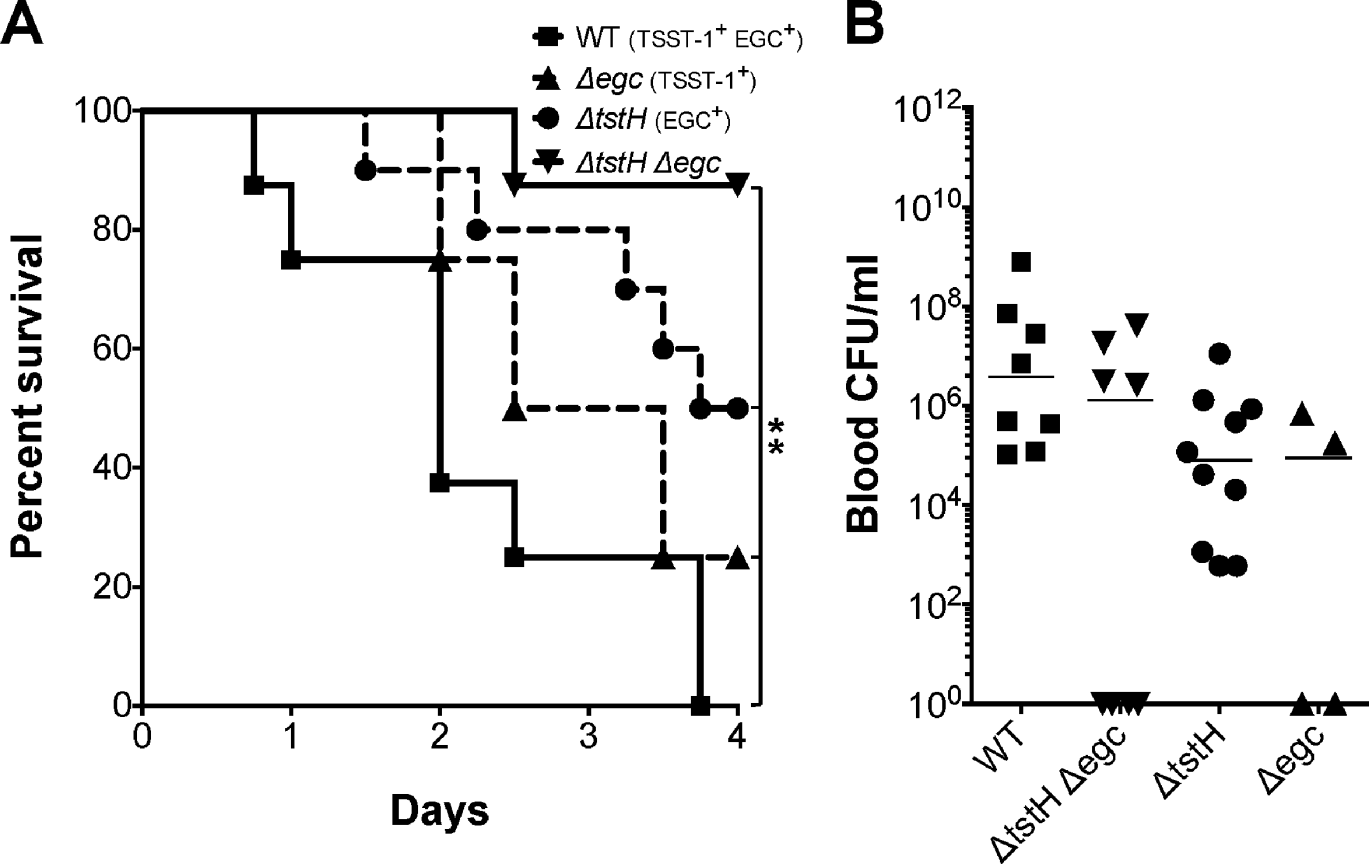
The *egc* SAgs and TSST-1 have differential effects on lethality associated with *S*. *aureus* strain MN8. (*A*) Percent survival of rabbits infected intravenously with 3–4 x 10^8^ CFU of wild-type MN8, MN8Δ*tstH*Δ*egc*, MN8Δ*tstH*, and MN8Δ*egc*. **P = 0.003, Log-rank, Mantel-Cox Test comparison of all conditions: wild-type MN8, MN8Δ*tstH*Δ*egc*, MN8Δ*tstH*, and MN8Δ*egc*. (*B*) Bacterial counts per milliliter of blood recovered from rabbits *post mortem*. Values not statistically significant (*P* = 0.2), one-way ANOVA and non-parametric, Kruskal-Wallis Test. *P* ≤ 0.05 is considered statistically significant.

Bacteria were injected intravenously at 3–4 x 10^8^ CFUs/rabbit, after mechanical damage of the aortic valves, and infection allowed to progress for a maximum of 4 days. Sixty percent of rabbits infected with wild-type MN8 succumbed to infection within two days, and by day 4 all rabbits succumbed (Fig 2A). Deletion of *tstH* resulted in 50% mortality (3 to 4 days post infection), while deletion of *tstH* and the *egc* significantly increased rabbit survival. To determine the contribution of *egc* SAgs to the observed phenotypes, we constructed an *egc-*deletion strain (MN8Δ*egc*). However, MN8Δ*egc* induced mortality comparable to that of wild-type MN8, where 80% of rabbits succumbed to infection by day 4 (Fig 2A). Wild-type MN8 and the three isogenic mutants were recovered from the blood at similar levels (Fig 2B), suggesting that bloodstream survival differences among infecting strains were not the major determinant of the differences in virulence observed.

**Fig 3:**
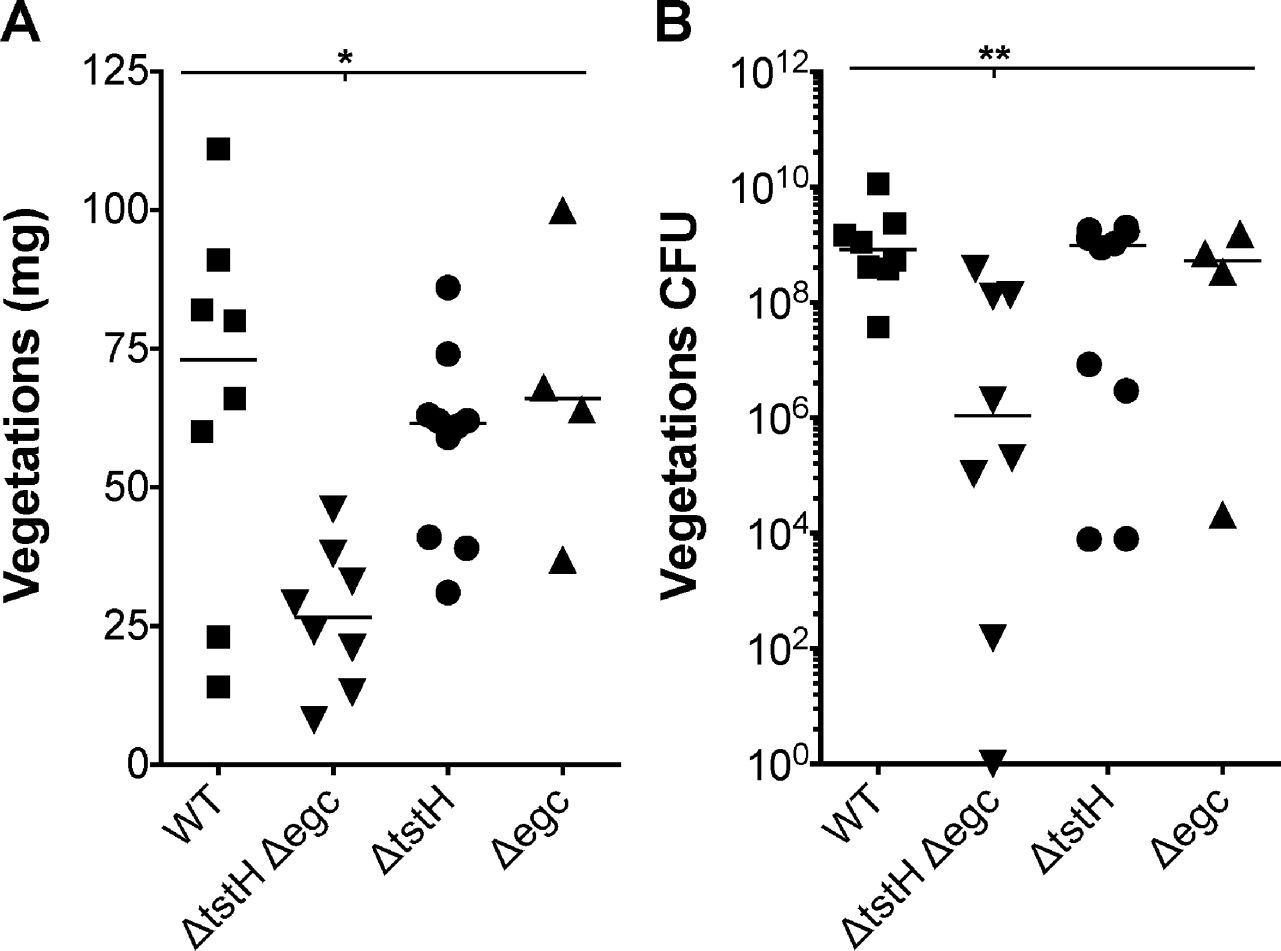
The *egc* SAgs and TSST-1 are involved in vegetation formation during IE. (*A*) Total weight of vegetations dissected from aortic valves after intravenous inoculation of 3–4 x 10^8^ CFU of wild-type MN8, MN8Δ*tstH*Δ*egc*, MN8Δ*tstH*, or MN8Δ*egc*. (*B*) Bacterial counts recovered from vegetations shown in panel A. *P = 0.01, **P = 0.03, one-way ANOVA and non-parametric, Kruskal-Wallis test. Horizontal lines represent the median. *P* ≤ 0.05 is considered statistically significant.

**Fig 4:**
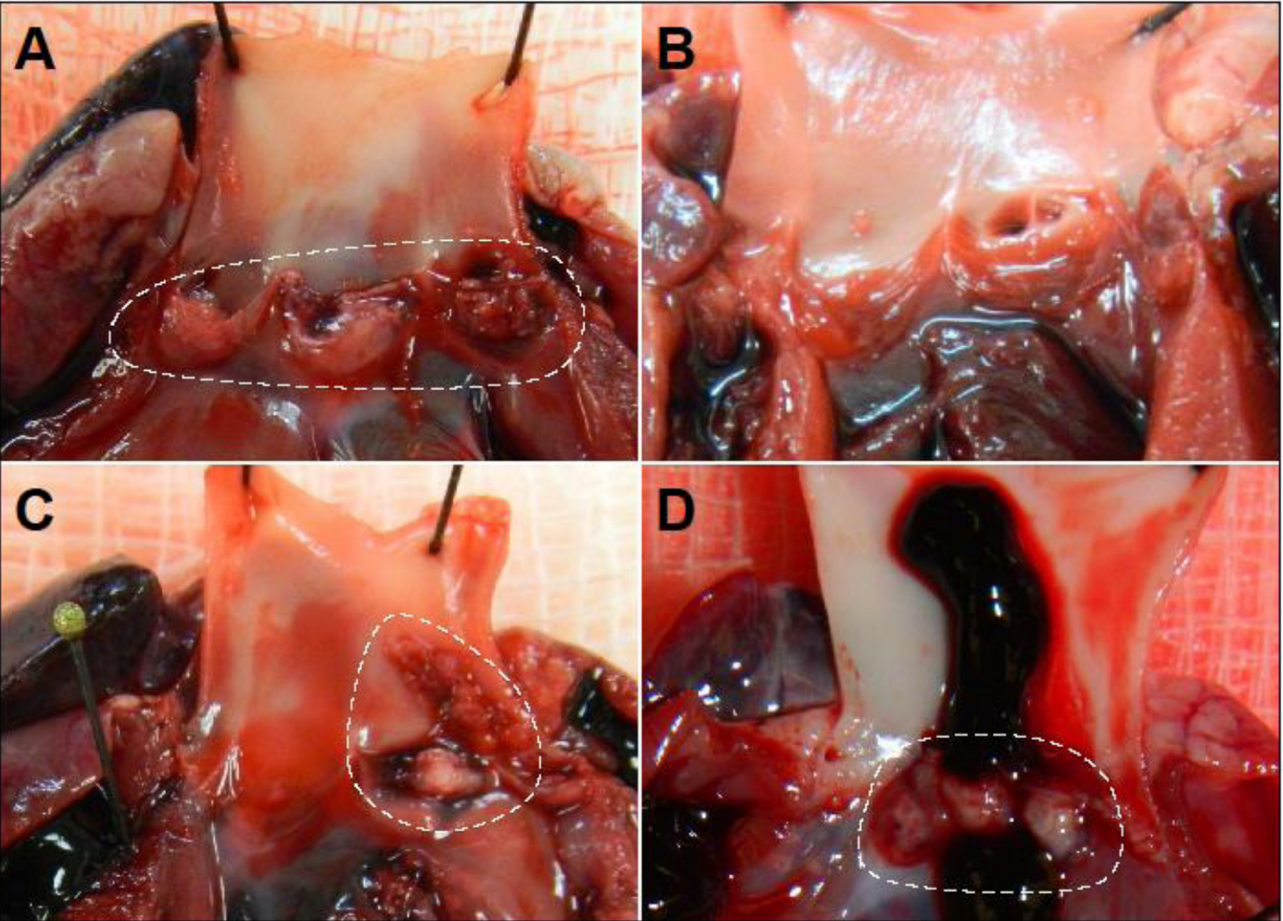
Deletion of the *egc* and *tstH* from MN8 leads to decreased vegetation formation. Representative images of vegetative lesions from (*A*) MN8 wild-type, (*B*) MN8Δ*tstH*Δ*egc*, (*C*) MN8Δ*tst*, and (*D*) MN8Δ*egc*.

Rabbit hearts demonstrated that deletion of either *tstH* or the *egc* resulted in vegetations similar to the wild-type strain (Figs 3 and 4). MN8 forms large vegetations on valve leaflets that associate with blood clots (Fig 4A). Most vegetations produced by MN8 (6/8) weighed 60–111 mg, with 4 x 10^8^–1 x 10^10^ CFU recovered from total vegetations (Fig 3). Even small vegetations, weighing 14 and 23 mg, had bacterial loads of 3.8 x 10^7^ and 1 x 10^9^ CFU respectively. MN8Δ*tstH* and MN8Δ*egc* produced vegetations averaging 58 mg and 67 mg in weight, respectively, with averages of 9 x 10^8^ and 7 x 10^8^ CFU recovered from vegetations, values similar to those of wild-type (Figs 3 and 4C and 4D). However, rabbits infected with MN8Δ*tstH*Δ*egc* developed small vegetations, where 2/8 vegetations weighed 8 and 13 mg (one vegetation was sterile and the other contained 150 CFUs), and 6/8 weighed 21–46 mg, with 2 x 10^5^–3.8 x 10^8^ CFUs, bacterial burdens significantly lower than wild-type (Figs 3 and 4B). Taken together, the data suggest that TSST-1 plays an early, critical role in lethality, while *egc* SAgs contribute to lethality with progression of IE. These results provide evidence for the important contribution of prevalent SAgs to development and severity of IE, and demonstrate that the *egc* SAgs are important factors in pathogenesis.

### TSST-1 and *egc* SAgs enable vegetation production in a SAg-negative strain

**Fig 5:**
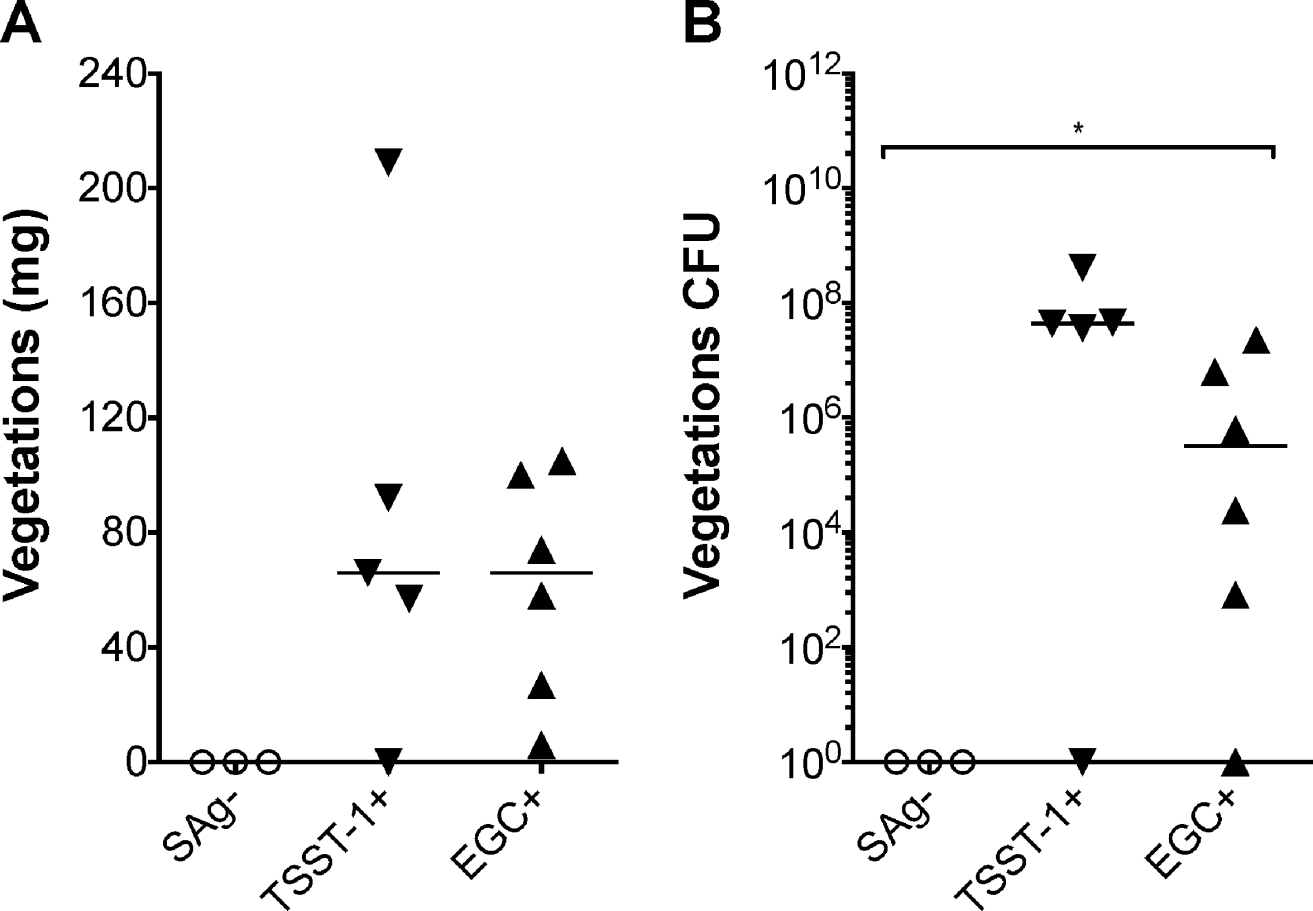
Expression of the *egc* or *tstH* leads to vegetation formation. (*A*) Total weight of vegetations dissected from aortic valves after intravenous inoculation of 2 x 10^9^–4 x 10^9^ CFUs of RN450 (SAg- empty vector), RN450 expressing TSST-1, or RN450 expressing the *egc* SAgs. (*B*) Bacterial counts recovered from aortic valve vegetations shown in panel A. *P* ≤ 0.05 is considered statistically significant. Horizontal lines represent the median.

**Fig 6:**
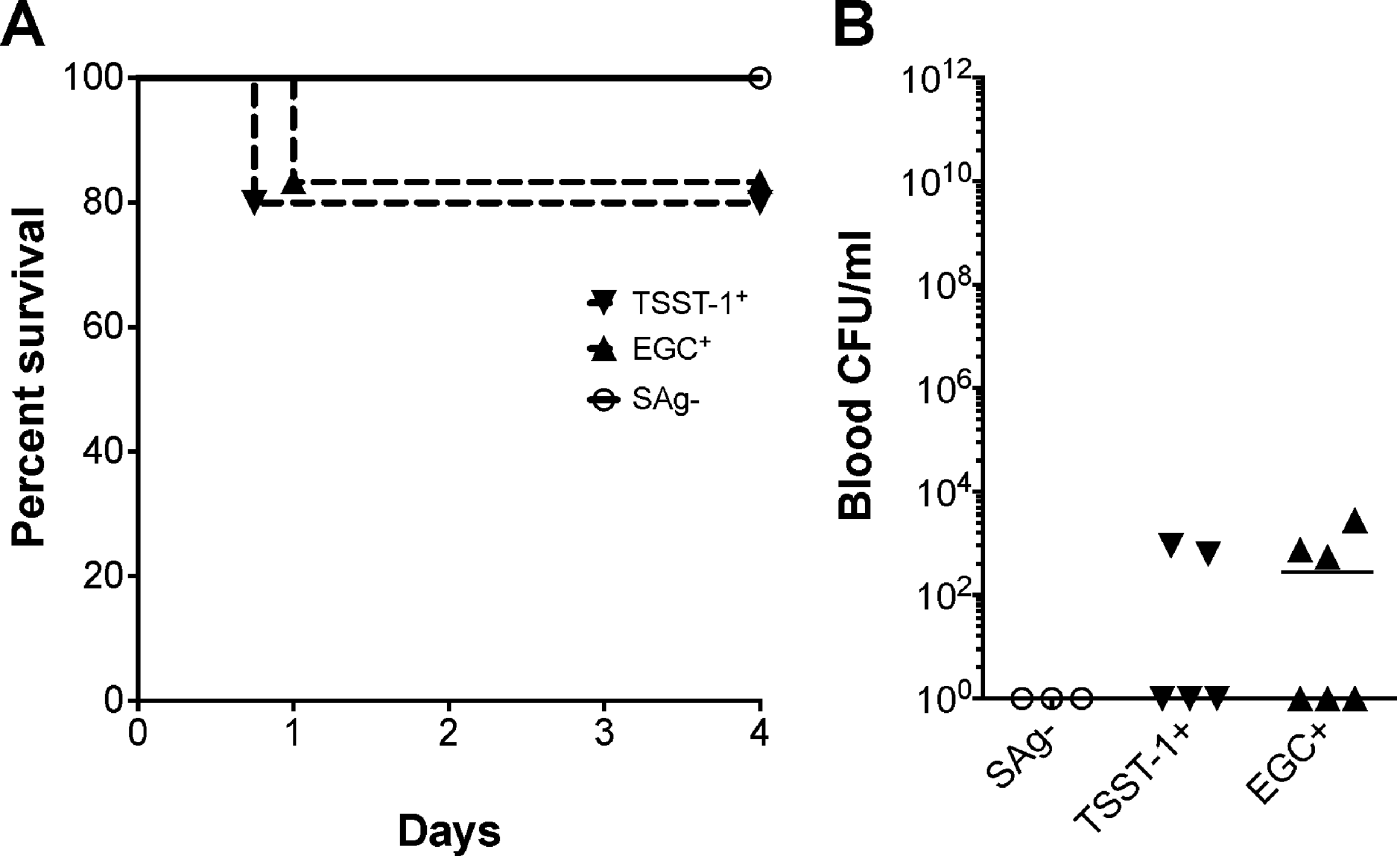
Lethality of RN450 expressing TSST-1 or the *egc* SAgs in the rabbit model of IE and sepsis. (*A*) Percent survival of rabbits infected intravenously with 2 x 10^9^–4 x 10^9^ CFU of RN450 (SAg-), RN450 expressing TSST-1, or RN450 expressing the *egc*. (*B*) Bacterial counts per milliliter of blood recovered from rabbits *post mortem*. *P* ≤ 0.05 is considered statistically significant.

An additional approach to test the role of TSST-1 and *egc* SAgs directly in development of *S*. *aureus* endocarditis is to infect rabbits with a laboratory strain lacking SAgs (RN450) complemented with either *tstH* or *egc*. RN450 strains ectopically expressing either TSST-1, *egc* SAgs, or harboring the empty vector were constructed and tested. RN450 (empty vector control) failed to induce vegetation formation (Fig 5). Furthermore, rabbits infected with RN450 survived to the end of the study (day 4), at which point they appeared to have cleared the infection, as indicated by bacterial counts in the blood below the limit of detection (Fig 6). However, infection with RN450 complemented with either TSST-1 or *egc* SAgs led to IE (Fig 5), resulting in ~20% mortality (Fig 6). Three of the five rabbits infected with the RN450 TSST-1^+^ strain developed vegetations weighing on average 72 mg and contained 3.6 x 10^7^–4.6 x 10^7^ CFUs, one rabbit had a massive vegetation (209 mg, 40 x 10^7^ CFUs), and one rabbit did not develop vegetations. All rabbits infected with the RN450 *egc*^*+*^ strain developed vegetations; most (4/6) weighed ~80 mg and contained 2.4 x 10^4^–2.3 x 10^7^ CFU, while the smallest vegetation, weighing 6 mg, was sterile (Fig 5). These results provide evidence that production of SAgs is sufficient to allow an otherwise avirulent strain to cause IE.

### The *egc* proteins have varying capacities to induce vegetations in infective endocarditis

Our studies demonstrated that the *egc* induced development of IE but did not address if each of the proteins within the *egc* have the capacity to promote vegetation formation. To address this, each of the *egc* genes was expressed ectopically in *S*. *aureus* RN450 and protein expression characterized by Western immunoblot. The amounts of each protein as tested by Western immunoblotting, from overnight cultures at stationary phase, were in order as encoded on the operon: SE*l*O 59 μg/ml; SE*l*M 58 μg/ml; SEI 25 μg/ml; SE*l*U 55 μg/ml; SE*l*N 5 μg/ml; and SEG 28 μg/ml.

**Fig 7:**
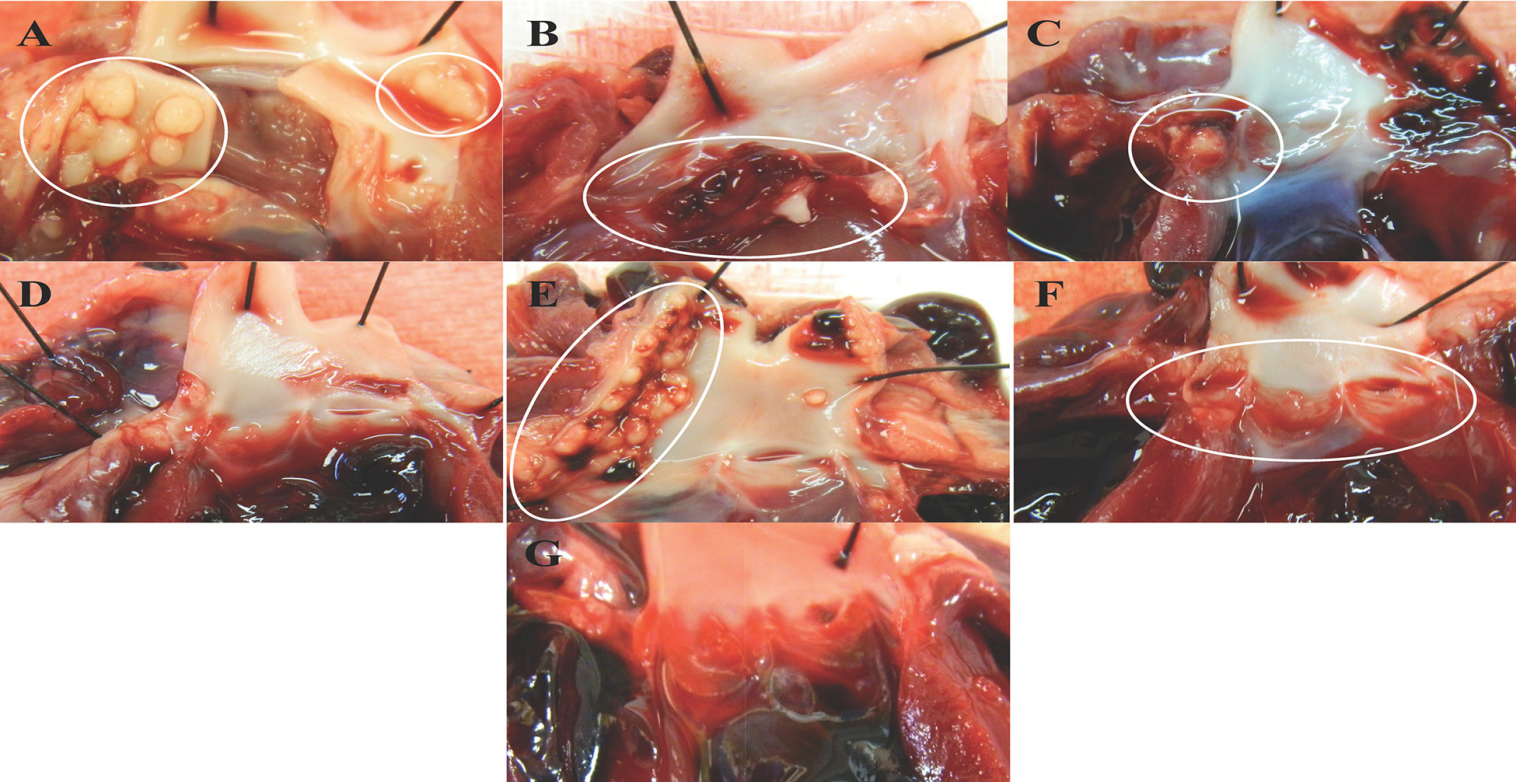
Individual *egc* proteins have the capacity to induce IE in RN450. (A) SE*l*O; (B) SE*l*M; (C) SE*l*U; (D) SEG; (E) SEI; (F) SE*l*N; (G) Vector control.

**Fig 8:**
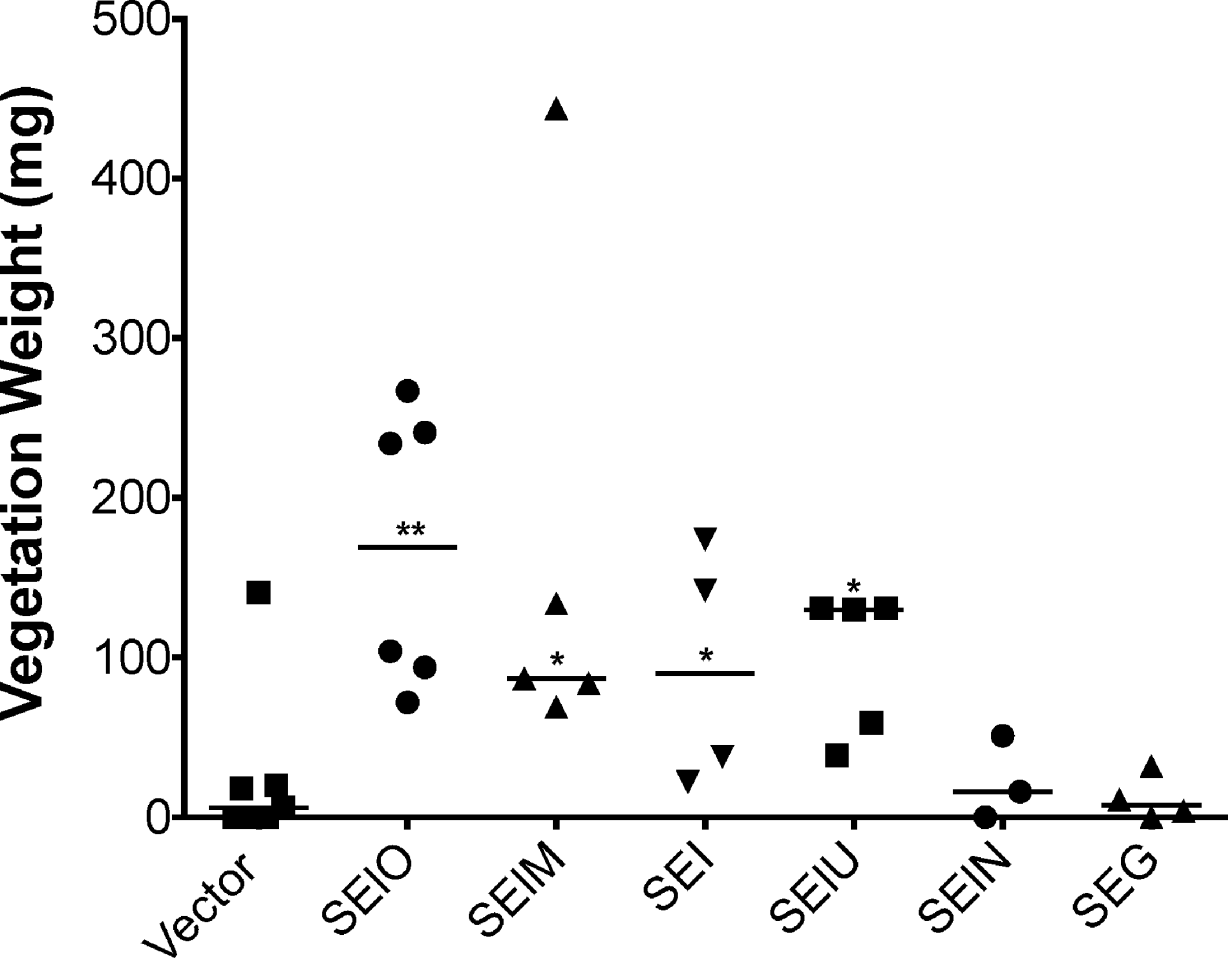
*egc* proteins differ in their ability to induce vegetations. Expression of SE*l*O, SE*l*M, SE*l*U, and SEI leads to vegetations. Expression of SEG and SE*l*N does not lead to development of vegetations. Horizontal lines represent the median. *P ≤ 0.05, **P ≤ 0.01, Mann-Whitney test. *P* ≤ 0.05 is considered statistically significant.

SE*l*M, SE*l*O, SE*l*U, and SEI individually led to vegetation formation in experimental IE when expressed in the SAg negative strain (Fig 7A–7C). SE*l*O, SE*l*M, and SE*l*U gave rise to the largest vegetations, with an average weight of 168 mg, 164 mg, and 98 mg respectively (Fig 8). Interestingly, SEG and SE*l*N were deficient in vegetation formation (Fig 7D and 7F and Fig 8), which may result from reduced protein production or instability. However, SEI and SEG strains differ in ability to form vegetations with SEI high and SEG low, despite both being produced at the same concentrations *in vitro*.

A principal feature of SAgs is resistance to trypsin digestion. We performed trypsin digestion assays to determine whether SE*l*N and SEG stability was impacting their ability to induce vegetation growth but they were shown to be stable (data not shown). All SAgs remained stable to trypsin treatment over a 24 hr test period. Interestingly, *in vivo* expression of the *egc* proteins did not result in splenomegaly, even in the presence of large vegetations on the heart valves, indicating the *egc* effects remain localized with minimal or no systemic involvement.

### Intergenic regions of the *egc* contain promoter elements

**Fig 9:**
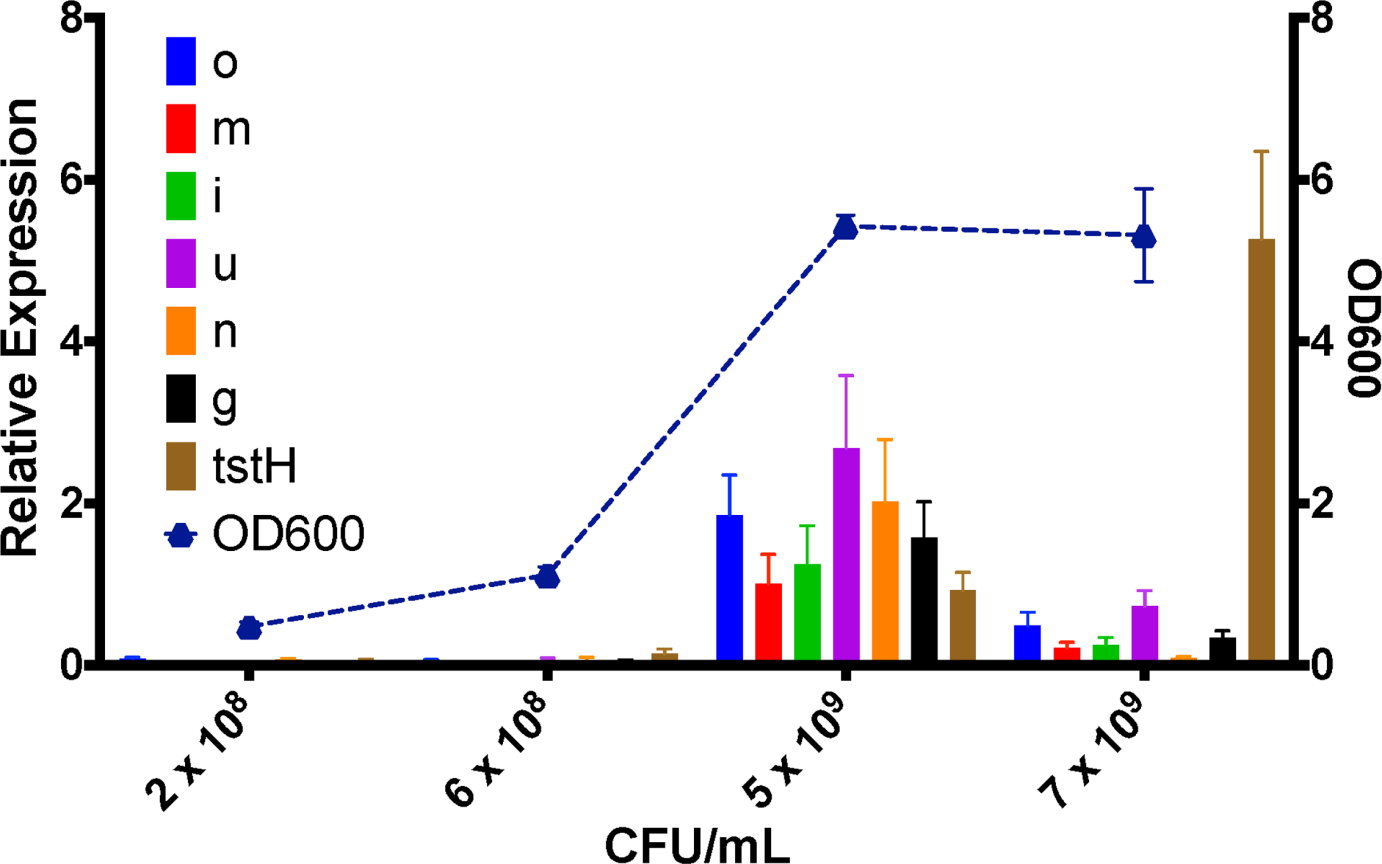
The *egc* SAg genes are expressed in *S*. *aureus* strain MN8. Expression of the *egc* transcript is represented in relation to cell density (OD 600 nm wavelength). Data are represented as the relative expression of each gene normalized to *gyrB* expression ± standard error of the mean.

**Fig 10:**
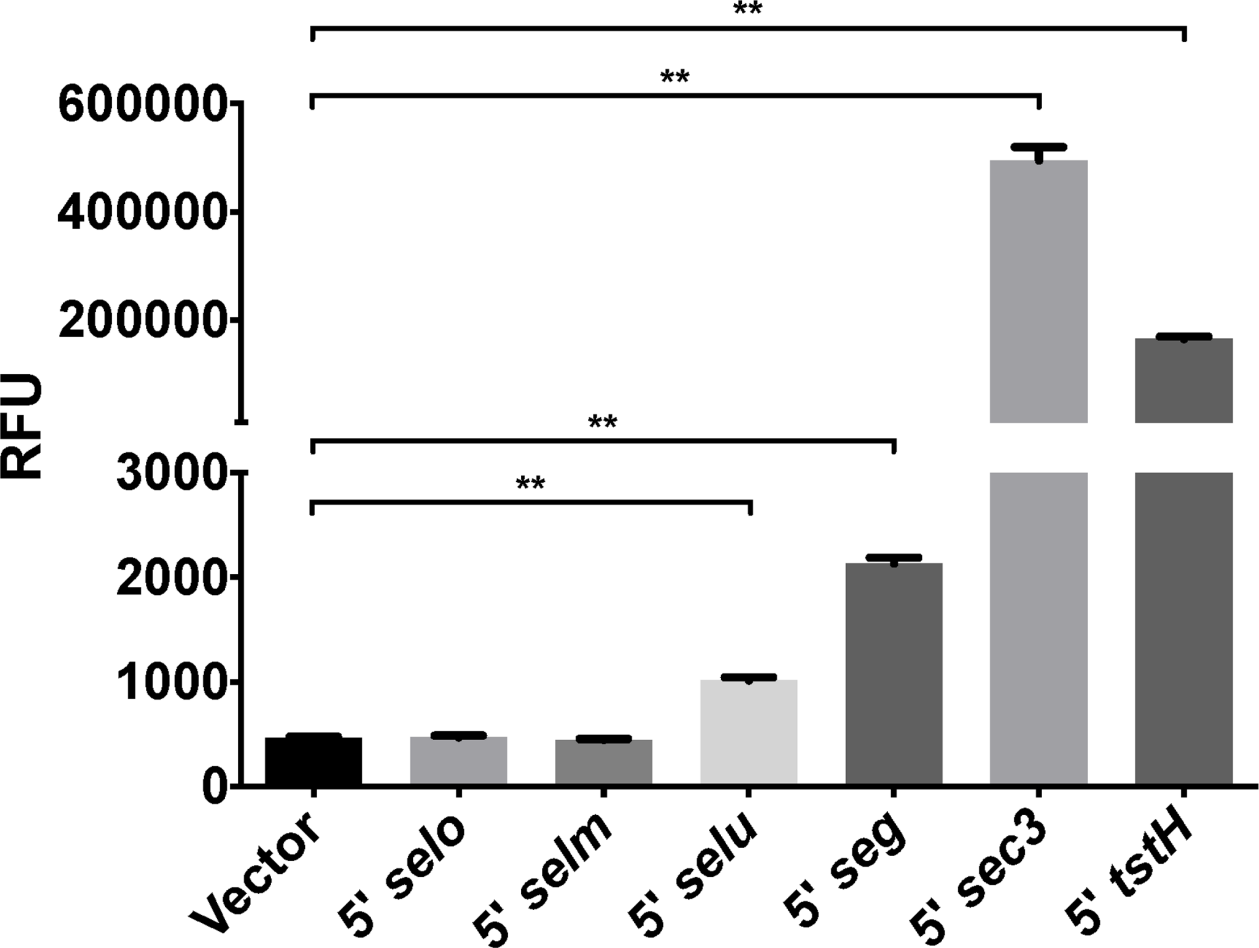
Fusions of 5′ *selu* and *5*′ *seg* regions of the *egc* induce GFP expression in RN4220. GFP expression as measured by relative fluorescence units (RFU) indicates 5′ *selu* and 5′ *seg* induce expression of GFP in RN4220 indicating potential promoter elements within the *egc*. Data displayed are means ± SEM, **P ≤ 0.01, Mann-Whitney test. *P* ≤ 0.05 is considered statistically significant.

qRT-PCR analysis indicated differential gene expression of the *egc* (Fig 9), which prompted us to investigate whether putative promoters were within the *egc* intergenic regions. The 5′ intergenic regions upstream of *selo*, *selm*, *selu*, and *seg* were fused to *gfp* (GFP) and assayed for fluorescence in *S*. *aureus* RN4220. *gfp* fused to *sec3* or *tstH* promoters were used as expression controls. Fluorescence was observed in the 5′ *selu* and *seg* constructs, which suggests that there may be promoter elements present in these regions (Fig 10). This is in agreement with the qRT-PCR showing higher expression of *selu* and *seg* in comparison to other genes in the *egc*.

### Human IVIG plus vancomycin reduces the severity of infective endocarditis

**Fig 11:**
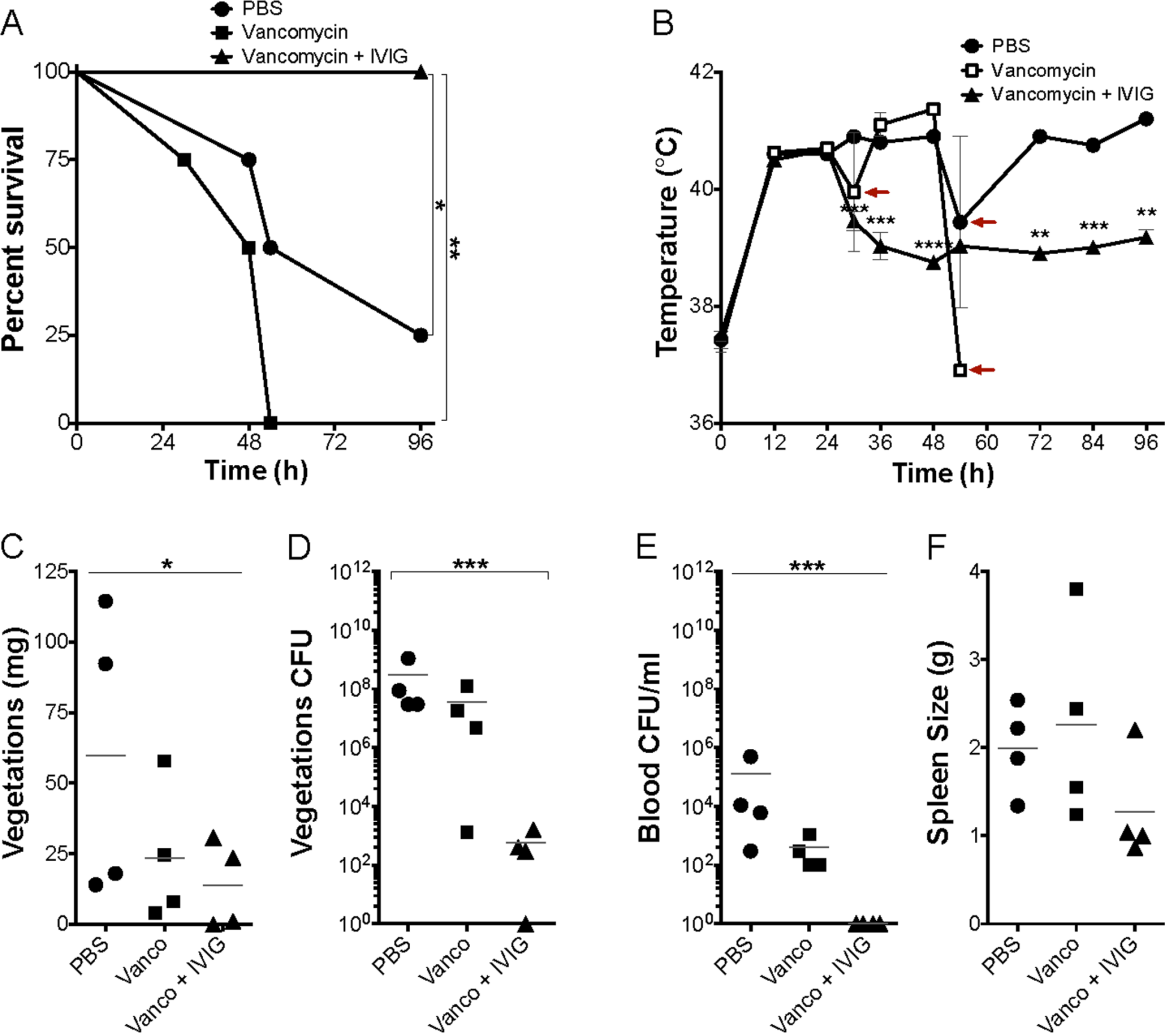
Vancomycin and IVIG treatment protects rabbits from lethality, vegetation formation, and bacteremia. Percent survival of rabbits infected intravenously with 2–4 x 10^8^ CFU/rabbit of *S*. *aureus* MN8 and treated with vancomycin alone, vancomycin plus IVIG or PBS 24 hrs post infection. *P* value determined with log-rank, Mantel-Cox test. (B) Rabbit temperatures over time. Red arrows indicate time points when temperature of some rabbits drops as they succumb to toxic shock. (C) Total weight of vegetations dissected from aortic valves and (D) bacterial counts recovered from vegetations; significance only between PBS and vancomycin plus IVIG treatment. (E) Bacterial counts per milliliter of blood recovered from rabbits *post mortem* and (E) splenomegaly resulting from *S*. *aureus* bacteremia. *P* value determined with one-way ANOVA and Dunnett’s multiple comparison test. (C-F) Horizontal lines represent mean values. *P ≤ 0.05, **P ≤ 0.01, ***P ≤ 0.001. *P* ≤ 0.05 is considered statistically significant. No *P* value means no statistical significance.

Patients with IE and sepsis are treated with antibiotics and supportive care. Previous studies have shown that IVIG reduces TSS severity caused by SAgs, most likely through neutralization of superantigenicity [42]. We tested the ability of IVIG co-administration with *S*. *aureus* MN8 to prevent development of IE and sepsis. IVIG prevented lethality (0/3 succumbed over 4 days) but did not completely prevent formation of vegetations (mean total vegetation weights = 4 mg), compared to PBS treated animals (3/3 succumbed on day 2; total vegetation weights = 8.5 mg); delaying administration of IVIG by 24 hr also prevented lethality in rabbits (0/3 succumbed), whereas delaying vancomycin by 24 hr did not prevent death (0/3 survived). These preliminary data suggested that the use of IVIG in combination with antibiotics such as vancomycin may be effective at reducing IE severity and lethal sepsis. Thus, an experiment was performed where rabbits in the usual IE and sepsis protocol were infected with *S*. *aureus* MN8, left untreated for 24 hr, and then administered PBS, vancomycin alone, or vancomycin plus IVIG for up to 24 hr (Fig 11). Significant differences in survival between rabbits receiving vancomycin plus IVIG was observed compared to rabbits receiving vancomycin alone or PBS (Fig 11A). It usually requires at least 24 hr for vancomycin alone to have an *in vivo* effect on *S*. *aureus*, and this likely explains why vancomycin alone did not improve survival. Additionally, IVIG plus vancomycin significantly reduced fevers in animals compared to vancomycin alone or PBS (Fig 11B). Total vegetation size in IVIG plus vancomycin animals was significantly reduced compared to animals receiving vancomycin alone or PBS, but was modestly reduced compared to animals receiving vancomycin alone (Fig 11C). However, rabbits treated with vancomycin plus IVIG had a five-log reduction in colony counts recovered from vegetations, bacterial counts in the blood below the limit of detection, and 3/4 rabbits exhibited no splenomegaly (Fig 11D–11F). Finally, 0/4 rabbits treated with IVIG plus vancomycin developed strokes, whereas cumulatively 4/8 rabbits receiving either vancomycin alone or PBS alone developed strokes. Altogether, these results indicate that IVIG enhanced the efficacy of vancomycin treatment.

## Discussion

*S*. *aureus* infections affect >500,000 people in the U.S. yearly [3]. Recently, it has been shown that SAgs are critical contributors to the pathophysiology of *S*. *aureus* pneumonia, IE, and sepsis [43, 44]. SAgs are thought to act primarily by complicating infections with development of TSS. TSS is the systemic manifestation of superantigenicity, where SAgs and the induced cytokine storm reach the bloodstream causing fever and capillary leak. Without effective treatment, hypoperfusion leads to multi-organ dysfunction and death. These effects appear to be the targets of neutralization with IVIG [42]. While it is clear that TSS results from infections with TSST-1, SEB, or SEC-producing strains [45], there are other SAgs variably encoded in *S*. *aureus* that may contribute to disease development. Here, we addressed the contribution of *egc* SAgs compared to TSST-1 in development of IE with sepsis. This approach led to three major findings: 1) *egc* SAgs and TSST-1 are important for vegetation formation on aortic valves, providing clear evidence of the significant role of SAgs in the etiology of IE, 2) *egc* SAgs most likely act locally to cause tissue pathology (heart vegetations), and 3) TSST-1 is uniquely associated with systemic toxicity and therefore, leads to early lethality and likely stroke development in IE.

Other researchers cite that advances in cardiovascular medicine achieved in the last decades have failed to improve IE outcomes, with mortality remaining high [4, 29, 46, 47]. *S*. *aureus* is the leading cause of IE in the developed world [4, 29, 46, 47]. Recent epidemiological studies highlighted the high prevalence of specific SAg genes in *S*. *aureus* strains from IE patients, specifically TSST-1, SEC, and *egc* SAgs [32, 33]. In this study, we used MN8, a USA200 isolate that encodes both *tstH* and the *egc*, to determine the contribution of SAgs highly represented in IE isolates to disease severity and progression, and furthermore to characterize the elusive role of the *egc* SAgs in *S*. *aureus* pathogenesis.

We demonstrate that concomitant deletion of *tstH* and the *egc* significantly reduces vegetation size and increases survival. However, deletion of *tstH* or the *egc* individually results in development of vegetations, with differential effects on survival. *tstH* deletion leads to survival similar to rabbits infected with MN8Δ*tstH*Δ*egc*, while *egc* deletion leads to lethality resembling that of wild-type MN8. A possible explanation is that TSST-1 (150 μg/ml *in vitro*) is produced at much higher levels than *egc* SAgs and hence, contributes to increased mortality through development of TSS and/or rapid progression of IE with development of lethal complications such as heart failure, strokes, and metastatic infections. Neutralization of TSST-1 is the suggested mechanism by which the combination of IVIG plus vancomycin reduced lethality and strokes in our studies. Interestingly, while deletion of *tstH* increases survival, it still results in large vegetations, leaving rabbits susceptible to complications and death associated with IE. Therefore, IE progresses even without the systemic or local effects of TSST-1 in the presence of *egc* SAgs.

Of importance, expression of either *tstH* or the *egc* in *S*. *aureus* RN450 results in development of IE. RN450 contains multiple mutations that negatively affect expression of surface proteins, stress responses, and exoprotein production [48]. RN450 is therefore avirulent in the rabbit model of IE and sepsis. However, in this deficient strain, ectopic production of *egc* SAgs or TSST-1 allows valve colonization and vegetation formation. These results demonstrate that TSST-1 and *egc* SAgs contribute independently to development of IE. We conclude that *egc* SAgs and TSST-1, which are encoded in the majority of IE isolates, are important contributors to the etiology and fatal outcomes characteristic of *S*. *aureus* IE. A decrease in morbidity and mortality due to *S*. *aureus* IE may be feasible with standard of care that incorporates SAg neutralizing agents, such as IVIG [20] or SAg production inhibitors [49] to allow antimicrobial therapy to have full effects.

We further characterized the *egc* by exploring the ability of individual proteins of the *egc* to cause IE. The findings of this study underscore the importance of SAgs in development and progression of IE and specifically identify that the proteins of the *egc* have varying capacities to induce vegetation formation in IE. This variation is interesting given that many of the *egc* SAgs belong to the same SAg families (i.e., SEG and SE*l*U or SE*l*O and SE*l*N) yet differ drastically in IE development. *In vitro* trypsinization assays did not reveal differences between SEG and SE*l*U or SE*l*O and SE*l*N in sensitivity to preoteolytic cleavage. However, we cannot exclude the possibility that the proteins have different sensitivities to *S*. *aureus* or host proteases, which would negatively impact their ability to induce vegetations. These results refute claims that the presence of any SAg will lead to similar disease or that all SAgs are equally inflammatory. The *egc* SAgs are present in up to 90% of *S*. *aureus* strains isolated from IE patients, and our results show they are capable of causing IE [32, 33]. Studies have labeled these genes as markers for *S*. *aureus* classification, dismissing their potential contribution to disease development [32, 33]. Our studies directly link the *egc* SAgs with vegetation formation in IE and clarify that they are important virulence factors in disease.

Little is known about the regulation of the *egc* SAgs. Previous studies indicated that sigma-B may regulate expression by interaction with the 5′ region of the first gene of the *egc*, *selo* [50]. Expression profiling of the *egc* obtained by qRT-PCR indicated that there may be differences in gene expression within the *egc* and this led us to probe the intergenic regions to identify promoter elements. Fusion of the 5′ non-coding upstream region of *selo*, *selm*, *selu*, and *seg* to GFP identified putative promoters within the intergenic sequence upstream of *selu* and *seg*. Differential expression of the *egc* genes may impact disease development.

**Fig 12:**
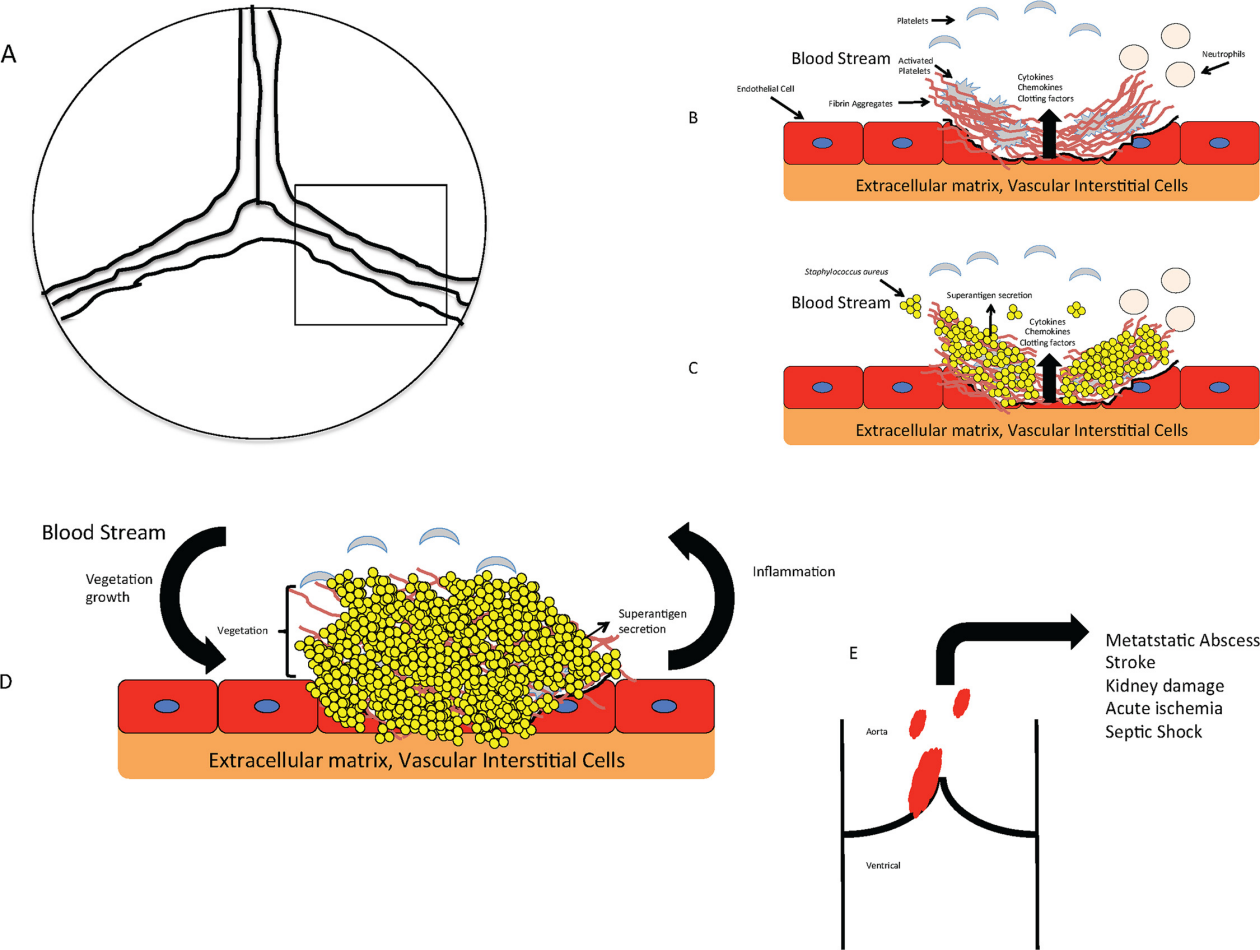
Model of *S*. *aureus* IE development on aortic valves. (*A*) Top down view of an aortic valve, with the represented boxed area used to provide an expanded model for the remaining panels. (*B*) Valve damage induces release of cytokines, chemokines, and clotting factors, leading to aggregation of host factors, including fibrin, erythrocytes, and platelets. (*C*) SAgs and exotoxins produced after valve colonization cause cytotoxicity, inhibiting healing of the damaged site and allowing bacterial growth (*D*) Bacterial persistence promotes further accumulation of host factors and vegetation expansion, inducing localized inflammation and increasing permeability of the aortic endothelium which allows SAgs access to the endocardium while recruiting immune cells. Superantigenicity within tissues is expected to promote immune dysfunction, chronic inflammation, and to support disease progression (*E*) Cross section of aortic valve. In large vegetations, pieces can detach leading to septic emboli that result in strokes and metastatic abscesses.

Superantigenicity at the tissue level, as observed on aortic valves, may occur by a mechanism similar to that described for menstrual TSS. During menstrual TSS, TSST-1 (and cytolysins) produced vaginally interact directly with the epithelium to induce inflammatory cytokines [51]. This initial inflammatory burst recruits immune cells to the subepithelium, providing accessibility to a sufficient pool of T cells and macrophages to elicit the cytokine storm characteristic of SAg-mediated toxinoses [52]. For development of IE, SAgs and cytolysins produced after colonization of valvular endothelia may cause cytotoxicity, inhibit healing of the damaged site, and promoting bacterial growth and accumulation of host factors. Localized inflammation may increase permeability of aortic endothelium allowing SAgs access to the endocardium while recruiting immune cells. In this instance, tissue superantigenicity would promote *S*. *aureus* persistence and vegetation growth on heart valves, as well as inducing cardiotoxicity and heart failure (Fig 12).

In conclusion, our study provides evidence for the significant role of SAgs in IE and demonstrates the role of *egc* SAgs in *S*. *aureus* life-threatening infections. To our knowledge this is the first report to directly associate the *egc* SAgs with IE and sepsis and more broadly to identify the local tissue effects of SAgs in development of infections. Of utmost importance, we provide evidence that IVIG plus an antibiotic is significantly more effective in reducing lethality and strokes associated with IE and sepsis than antibiotic treatment alone.
